# Comparative Evaluation of Fracture Resistance of Composite Core Buildup Materials: An In Vitro Study

**DOI:** 10.7759/cureus.63298

**Published:** 2024-06-27

**Authors:** Deepmala Pande, Niharika Benjamin, Vishakha Rani, Malik Hina, Shreya Haldar, Harni Nirmal, Shivani Gole, Md Sadique Ali

**Affiliations:** 1 Department of Prosthodontics, Hitkarini Dental College and Hospital, Jabalpur, IND; 2 Department of Public Health Dentistry, Hitkarini Dental College and Hospital, Jabalpur, IND; 3 Department of Public Health Dentistry, Dr. B.R. Ambedkar Institute of Dental Science and Hospital, Patna, IND; 4 Department of Prosthodontics, Saraswati Dental College, Lucknow, IND; 5 Department of Periodontics, Sardar Patel Post Graduate Institute of Dental and Medical Sciences, Lucknow, IND; 6 Department of Oral Surgery, Madha Dental College and Hospital, Chennai, IND; 7 Department of Dentistry, Rishiraj College of Dental Sciences and Research Centre, Bhopal, IND

**Keywords:** nanohybrid, fracture resistance, fiber-reinforced composite, nanofiller composite, core buildup materials

## Abstract

Aim

This study aimed to compare the fracture resistance of different materials used in composite core buildups, including conventional filler composite, nanofiller composite, and short fiber-reinforced composite (SFRC).

Methods

This in vitro study was conducted on 30 freshly extracted premolars. The teeth were treated using a uniform endodontic procedure, and Fiber Posts (REFORPOST, Angelus) were placed. The teeth were then divided into three groups and restored using different materials. Group 1 was restored using SFRC (everX Posterior, GC, Europe), Group 2 using microfiller composite (Te-Econom Flow, Ivoclar Vivadent), and Group 3 using nanofiller composite (Tetric N-Flow, Ivoclar Vivadent). The restoration materials were then light-cured for 40 seconds. The teeth were placed in a Universal Testing Machine (Instron) and a load was applied with a stainless-steel ball (4 mm diameter) until the tooth fractured. The fracture load for each tooth was recorded, and after the mechanical test, the experimental groups were examined for failure modes. Statistical analysis was performed using SPSS version 21.0 software. A one-way ANOVA test was conducted to compare more than two groups, followed by Tukey’s test for post hoc pairwise comparison.

Results

The mean fracture resistance of the microfiller composite (346.94±44.63) was the lowest among the three groups. When analyzed using Tukey's test at p<0.05, fracture resistance was significantly higher in the SFRC, followed by nanofillers and microfiller composites.

Conclusion

Due to the increasing demand for aesthetic restorations in recent years, composites have become important in modern restorative dentistry. The development and implementation of composite dental restorative materials rely on a comprehensive understanding of each composite component and consideration of methods for modifying each component. As a result, the findings of this study will be beneficial in determining which material to use based on specific cases.

## Introduction

Over recent decades, the demand for aesthetic dental restorations has escalated, resulting in increased utilization of composite materials in contemporary restorative dental procedures [1]. The development and application of these composite materials necessitate a thorough understanding of each composite component and the exploration of methods for their modification [2]. Resin-based composites, first introduced in the early 1960s, demonstrated superior mechanical properties compared to acrylics and silicates, exhibited reduced dimensional change upon setting, and displayed enhanced wear resistance, thereby augmenting their clinical performance [3].

Dental composites can be categorized based on the restorative procedure, filler particle size, curing modes, and clinical applications [4]. Advancements in composite technology have facilitated the creation of modern materials with improved durability, wear resistance, and aesthetics that mimic natural teeth. Nanotechnology has significantly contributed to the enhancement of these materials through the control of filler architecture [3].

Microfilled composites are effectively utilized in anterior restorations, such as class 3 and class 5 cavities, and facial veneers, where aesthetics is the primary concern [5]. Nanohybrid composites are deemed the gold standard for posterior restorations due to their dense filler loading, exceptional optical properties, and improved mechanical properties. However, they experience polymerization shrinkage (1.6-7.1%), which induces contraction stresses potentially leading to microleakage and restoration failure [6].

The advent of fiber-reinforced composite (FRC) technology has expanded the use of composite resin materials in extensive preparations. The inclusion of fibers in the resin enhances resistance to crack propagation while reducing shrinkage and creep. Recently, short fiber-reinforced composite (SFRC) (polyethylene) has been introduced as a dental restorative composite resin capable of halting crack propagation and serving as a load-bearing barrier under substantial occlusal forces [7]. Composites are employed in a variety of restorations, including cavity and crown restoration materials, adhesive bonding agents, pit and fissure sealants, endodontic sealants, bonding of ceramic veneers, and as luting agents for cementation of crowns, bridges, and other fixed prostheses, as well as core buildup materials [5,7].

Prosthetic rehabilitation of endodontically treated teeth presents a challenge due to the loss of blood supply and dental lymph, and the calcification of peritubular dentin, resulting in brittleness [8]. When functional and parafunctional stresses are exerted on the occlusal table of the tooth, both the restoration and the tooth can fracture. Endodontically treated teeth with insufficient coronal tooth structure typically require a post and core to restore function [8,9].

Metal posts are no longer utilized due to their propensity to induce vertical root fractures. These posts have a high modulus of elasticity, resulting in stress transmission from the rigid post to the less rigid dentin [10,11].

FRC posts are considered a superior alternative to cast metal posts due to their higher retention rate and lower failure rates. Introduced in 1990, FRC posts have gained popularity among clinicians due to their physical properties such as high tensile strength and good fatigue resistance. Additionally, their modulus of elasticity is similar to that of dentin, enhancing their compatibility when combined with FRC posts [12]. Composite core buildup material is typically employed to restore the coronal portion of teeth and achieve retention and resistance form for the crown. The use of FRC post and core restoration has been reported to have a high success rate with a reduction in root fracture failure [13].

Contradictory results have been reported by different authors. Some authors [6,7,14-19] reported increased fracture resistance with SFRC, whereas others [20-22] reported promising performance with nanohybrid composite material. A few authors [23] reported no significant difference between SFR and nanohybrid composites. Studies [15] have been conducted to compare the fracture resistance of different composite core build-up materials in the anterior region. However, there is a relative scarcity of studies that have compared the fracture resistance of conventional filler composite, nanofiller composite, and SFRC core build-up materials in posterior teeth with fiber posts.

The objective of this study is to compare the fracture resistance of these three composite core buildup materials and identify the most fracture-resistant core buildup material. The null hypothesis posits that there is no difference in the fracture resistance of these three composite core buildup materials.

## Materials and methods

This study was conducted at the Department of Prosthodontics and Crown and Bridge, Saraswati Dental College and Hospital in Lucknow. Prior to the commencement of the study, ethical clearance was obtained from both the Institutional Research and Development Committee (IRDC) (Ref: SDC/IRDC/2018/MDS/26) and the Institutional Human Ethics Committee (IHEC) (Ref: SDC/IHEC/2018/MDS/26). Sample size estimation was performed using G Power software (version 3.0). The sample size was estimated for an ANOVA test (comparing the mean fracture resistance of more than two groups). A total minimum sample size of 30 (10 for each group) was deemed sufficient for an alpha of 0.05, a power of 95%, and an effect size of 0.77. The details of the groups compared in the study can be found in Table 1.

**Table 1:** Details of groups compared in the study.

Group	Post Materials	Core Build Up Materials
Group 1	Fiber Post (REFORPOST, Angelus)	Short Fiber Reinforced Composite (everX Posterior, GC)
Group 2	Fiber Post (REFORPOST, Angelus)	Microfiller composite (Te-Econom Flow, Ivoclar Vivadent)
Group 3	Fiber Post (REFORPOST, Angelus)	Nanofiller composite (Tetric N-Flow, Ivoclar Vivadent)

Thirty freshly extracted mature human maxillary premolars were selected for data collection. Only teeth without caries, abrasion, cavities, and fractures were included in the study. Teeth containing debris and soft tissue remnants were sterilized and stored in a 0.1% thymol solution at room temperature. Endodontic access cavities were prepared using a water-cooled diamond bur in a high-speed handpiece, and the entire pulp tissue was removed simultaneously. A size 15 K-file was placed into each canal until it was visible at the apical foramen, and a working length was established 1 mm shorter than the file. The canals were prepared up to the size of an F5 Protaper rotary file at the working length. After each instrument was used, the canals were irrigated with 3 mL of 2.5% sodium hypochlorite (NaOCl). The canals were then dried with paper points and filled with tapered gutta-percha and a resin-based root canal sealer (Enseal, Dentocare Pvt. Ltd.) using the lateral compaction technique.

**Figure 1:**
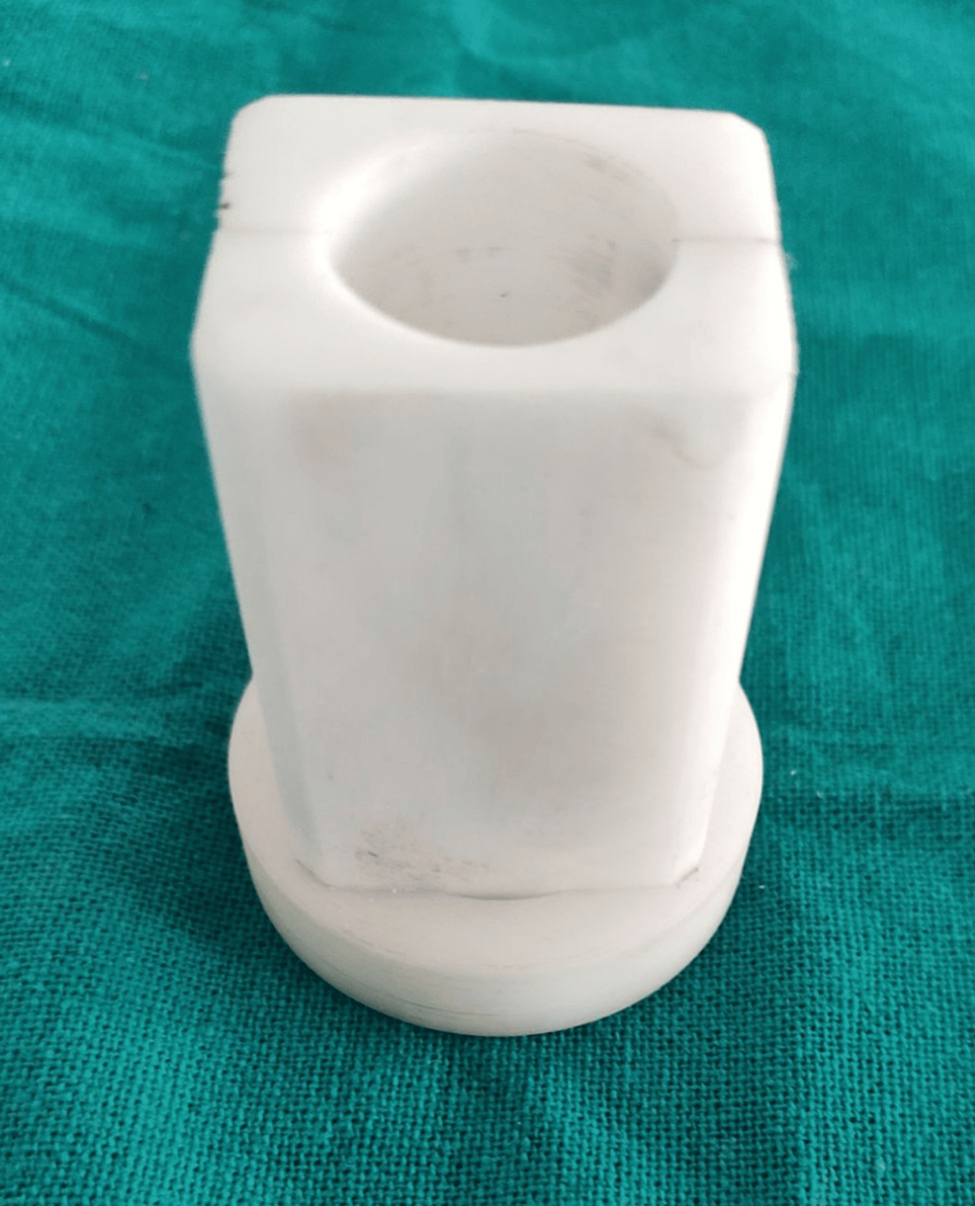
Teflon mold (5 cm x 2 cm).

The specimens were placed parallel to the long axis of the teeth in a Teflon mold measuring 5 cm in length and 2 cm in width (Figure 1).

**Figure 2:**
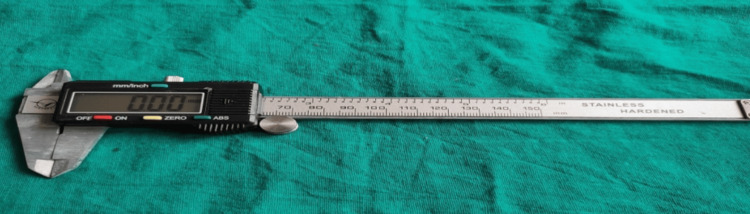
Digital Vernier calliper used for measurements.

The specimens were placed up to 2 mm below the cemento-enamel junction to imitate the alveolar bone. The palatal cusps were removed and cavity preparation was performed using a fissure diamond bur. The cavity floor was made perpendicular to the long axis of the tooth. The lengths and the largest buccopalatal and mesio-distal widths of the root were measured using a digital caliper to ensure that the cavity dimensions were accurate (Figure 2).

**Figure 3:**
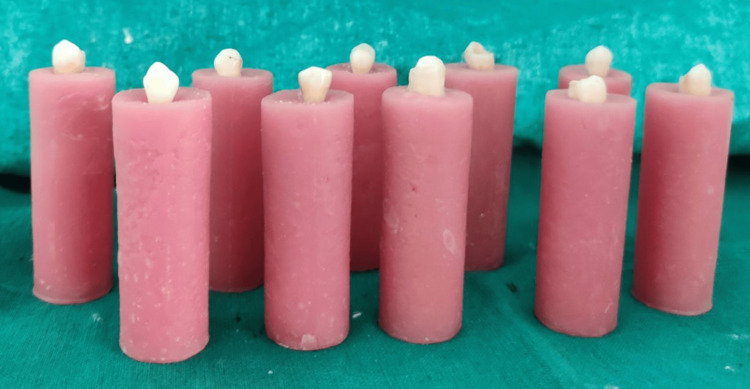
Prepared samples divided into three groups of 10 specimens each.

The teeth were randomly divided into three groups of 10 specimens each (Figure 3).

Prior to post placement, post spaces were cleaned with 2.5% NaOCl, then rinsed with distilled water, and dried with paper points. A self-etch adhesive (G-Bond, GC) was applied to the walls, thinned using a brush, and light-polymerized for 20 seconds. The entire fiber resin post was then light-cured for 40 seconds.

**Figure 4:**
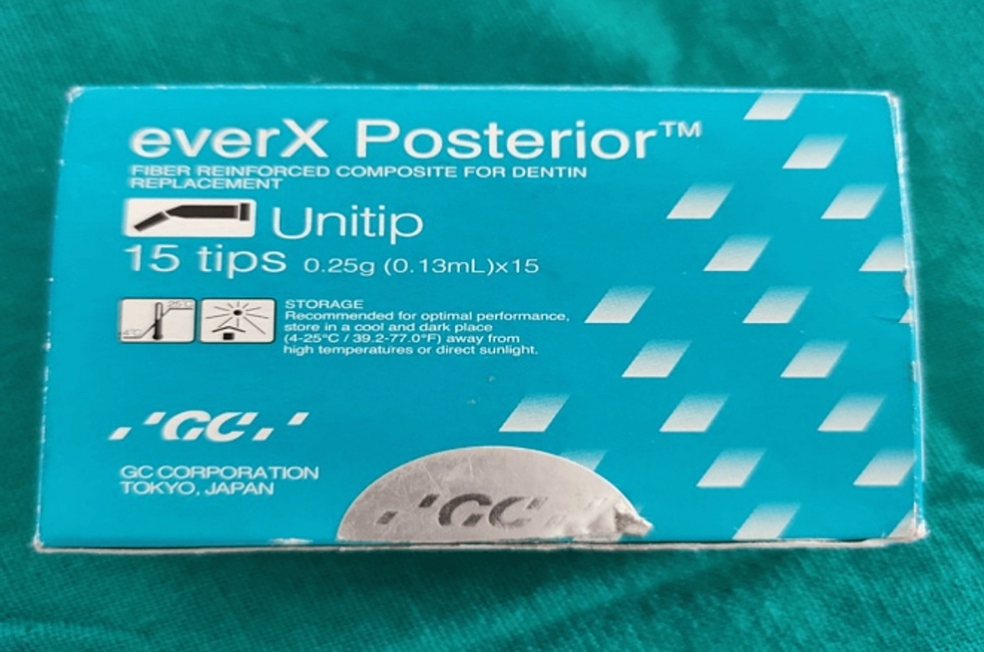
Short fiber-reinforced composite (everX Posterior).

In Group 1, SFRC resins (all SFRC), SFRC (EverX Posterior, GC) were used for the restoration of deep cavities and light-cured for 40 seconds. The coronal part was restored with SFRC and light-cured for 40 seconds from all directions (Figure 4).

**Figure 5:**
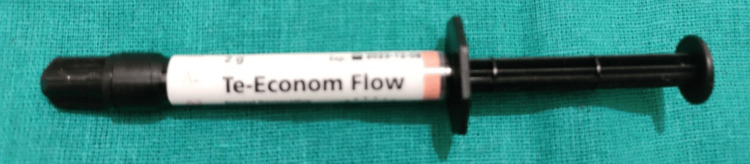
Microfiller composite (Te-Econom plus).

In Group 2 (all microfiller composite), microfiller composite (Te-Econom Flow, Ivoclar Vivadent) was used for the restoration of deep cavities and light-cured for 40 seconds. The coronal part was restored with microfiller composite and light-cured for 40 seconds from all directions (Figure 5).

**Figure 6:**
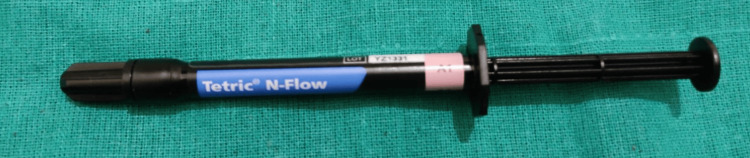
Nanofiller composite (Tetric N-Flow).

In Group 3 (all nanofiller composite), nanofiller composite (Tetric N-Flow, Ivoclar Vivadent) was used for the restoration of deep cavities and light-cured for 40 seconds. The coronal part was restored with nanofiller composite and light-cured for 40 seconds from all directions (Figure 6).

**Figure 7:**
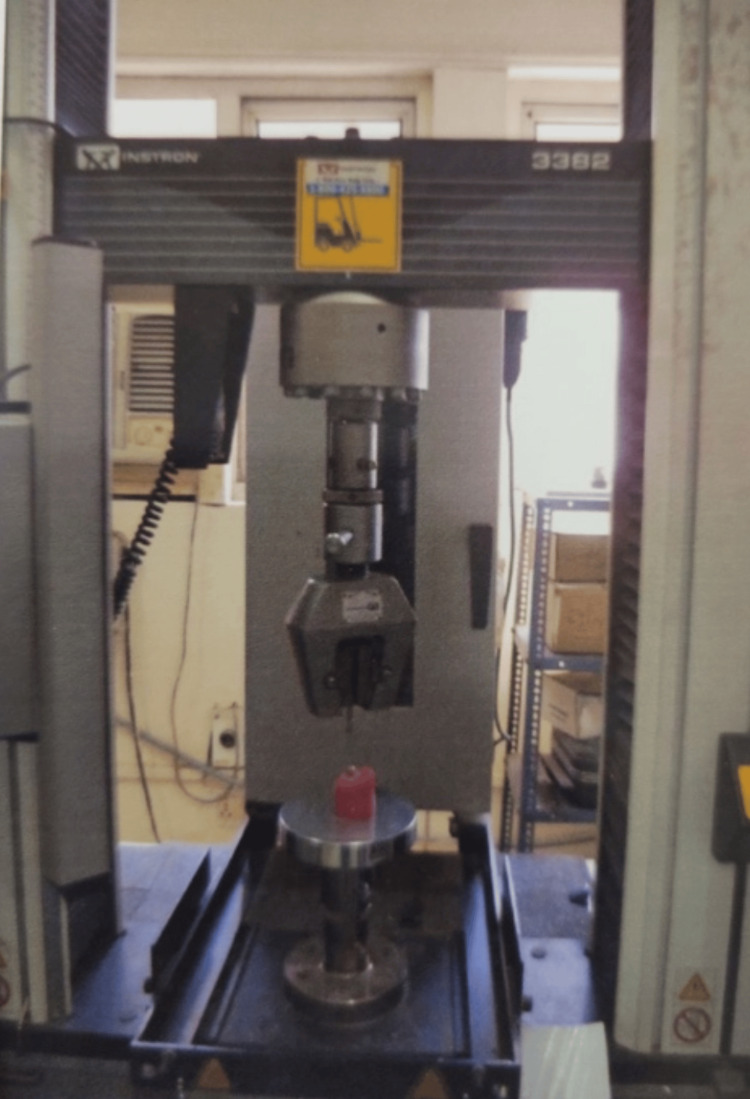
Specimen placed on Universal Testing Machine (UTM)-INSTRON.

All specimens were stored in normal saline at 37°C for a week. Specimens were placed into a Universal Testing Machine (Instron) with the long axis of the roots at an angle of 30° to the load direction (Figure 7).

The load was applied with a stainless-steel ball (4 mm diameter) at a crosshead speed of 1 mm/min until the fracture occurred. The loading site was the central fissure of the occlusal surface, and the fracture load for each tooth was recorded. After the mechanical test, the experimental groups were examined for failure modes.

The data for the standard fracture resistance test were obtained, and statistical analysis was performed using SPSS version 21.0 software.

## Results

The SPSS software (version 21.0) was utilized to analyze the data. The Shapiro-Wilk test was conducted to determine which variables followed a normal distribution. The results indicated that the data followed a normal distribution (p-value greater than 0.05). The p-values for SFRC, Microfiller, and Nanofiller were 0.795, 0.125, and 0.878, respectively. Therefore, a one-way ANOVA test was conducted to compare more than two groups, followed by Tukey’s test for post hoc pairwise comparison. It is worth mentioning that the level of statistical significance was set at a p-value less than 0.05.

Group-wise comparison of fracture resistance was performed. The mean fracture resistance of the SFRC was found to be 697.62 ± 20.40, for the microfiller it was 346.94 ± 44.63, and for the nanofiller composite, it was 439.16 ± 33.53 (Table 2).

**Table 2:** Group-wise comparison of fracture resistance.

	Mean	Std. Deviation	Std. Error	95% CI for Mean		Minimum	Maximum
				Lower Bound	Upper Bound		
Short-Fiber Reinforced Composite	697.6220	20.40936	6.45401	683.0220	712.2220	660.30	723.78
Microfiller composite	346.9460	44.63774	14.11569	315.0141	378.8779	240.60	399.00
Nanofiller composite	439.1690	33.53007	10.60314	415.1830	463.1550	385.87	488.00

A one-way ANOVA was performed. Overall, significant differences were observed in the fracture resistance of the three study groups as p < 0.05 (Table 3).

**Table 3:** One-way ANOVA (p<0.05).

	Sum of Squares	Df	Mean Square	F	Sig.
Between Groups	660922.306	2	330461.153	280.580	0.0001*
Within Groups	31800.022	27	1177.779		
Total	692722.329	29			

Post hoc pairwise comparisons using Tukey's test were performed. Significant differences were observed in the fracture resistance of the three study groups. Fracture resistance was found to be significantly higher in the SFRC, followed by nanofillers and microfiller composites when analyzed using Tukey's test at p < 0.05 (Table 4).

**Table 4:** Post-hoc pairwise comparison using Tukey's test (p<0.05).

	Mean Difference	Std. Error	Sig.	95% CI	
				Lower Bound	Upper Bound
Short-Fiber Reinforced Composite vs Microfiller	350.67600^*^	15.34782	0.0001*	312.6223	388.7297
Short-Fiber Reinforced Composite vs Nanofiller	258.45300^*^	15.34782	0001*	220.3993	296.5067
Microfiller vs Nanofiller	-92.22300^*^	15.34782	0001*	-130.2767	-54.1693

## Discussion

The prognosis of teeth treated endodontically is contingent not only on the treatment procedure but also on the quality of the coronal restoration [7]. The dentine in such teeth undergoes alterations in collagen cross-linking and subsequently dries over time. Consequently, a reduction in their modulus of elasticity is observed, often resulting in heightened fracture susceptibility compared to unrestored vital teeth [24].

In this study, extracted human maxillary premolars were utilized as test specimens, given that clinical observations have indicated that endodontically treated premolars are the most frequently fractured teeth. Premolars are subjected to both shear and compressive forces during mastication. The combination of these forces increases the likelihood of fractures, particularly in endodontically treated teeth where the structural integrity is already compromised [6,7]. As lingual cusps are most commonly fractured under compressive loading in clinical scenarios [7], the premolars were prepared similarly, with missing lingual cusps restored using various core build-up materials.

Previous research has indicated that endodontically treated teeth are prone to fractures [13]. To enhance the fracture resistance of these teeth, a range of materials have been employed for core build-up procedures. This study compared the fracture resistance of three composite core build-up materials: SFRC, microfiller composite, and nanofiller composite. The null hypothesis was refuted based on the study's findings, which revealed that the fracture resistance of SFRC was significantly higher than that of nanofiller and microfiller composites.

The findings of this study align with those of Bilgi PS et al., Gürel MA et al., and Garlapati TG et al. [6,7,14]. In the study by Gürel MA et al., the SFRC group showed the highest fracture resistance and had the highest number of restorable fractures, while endodontically treated teeth restored with everX posterior FRC demonstrated superior fracture resistance in the study conducted by Garlapati TG et al.

It was deduced that the short E-glass fiber in the short fiber composite induces cross-linking, thereby inhibiting and preventing crack propagation that typically originates from the restoration surface and acts as a load-bearing barrier under high occlusal forces.

Other research has concluded that placing fiber under composite restoration significantly enhances the fracture strength of endodontically treated teeth [13]. Therefore, the composite resin is intended for use in high-stress-bearing areas [6,7]. It has also been suggested that the maximum fracture resistance is exhibited by the FRC, following intact teeth [6].

Several authors [6,18] have concluded that the fracture resistance value of FRC restorative material surpasses that of nanohybrid resin composite [18]. They discovered that the FRC demonstrated higher fracture toughness and flexural strength, and a lower percentage of shrinkage strain than conventional and bulk fill resin composites. The strength of nanohybrid composite has been found to exceed other conventional core build-up materials like amalgam and resin-modified glass ionomer [20].

MI FIL, introduced in 2010, is a flowable nano-hybrid composite in which the ultrafine particulate filler, with a mean particle size of 200 nm, is homogeneously and densely dispersed. It possesses adequate strength and abrasion resistance, potentially making it beneficial for aesthetic posterior restorations [21].

However, some authors have contradicted the results of this study, reporting no significant difference between SFR and nano-hybrid composites [22]. This study focused on flowable-type nano-hybrid composites and paste-type composites, evaluating parameters such as retention, surface texture, anatomical form, marginal discoloration, marginal adaptation, secondary caries, and color matching. It involved real-world application by multiple operators, which introduces variability in technique and patient factors.

The filler percentages of microfiller composites are significantly lower than those of nanofiller and SFRCs. Manufacturers claim that incorporating nanoparticles in resin composites enables the production of materials with increased filler load and improved physical and mechanical properties without increasing their viscosity. Research conducted by several authors has demonstrated that the modulus of elasticity of microfiller composites is significantly lower than that of nanofiller composites, which exhibit the lowest mechanical properties by a considerable margin [19]. The selection of core material must incorporate an understanding of material properties, and no material may be considered ideal or capable of truly replacing lost tooth structure. Clinicians must possess a clear understanding of the mechanical properties of materials to achieve the best clinical outcome [19].

Limitations of the study

The limitation of this study is its in vitro nature, which cannot fully replicate oral conditions. Additionally, the focus on maxillary premolars means the findings might not directly apply to other types of teeth, such as molars or incisors, which have different structural and functional characteristics. Another limitation is the type of test used. A Universal Testing Machine (UTM)-INSTRON was used in this study, which applied only compressive force to the tested specimens under static load at a single point in a monostatic pattern. This does not apply tensile and shear forces that represent intraoral conditions.

The study emphasizes that SFRC has significantly higher fracture resistance compared to Microfiller and Nanofiller composites. This suggests that SFRC is superior for core buildup in endodontically treated maxillary premolars. Clinically, this means that SFRC can be particularly useful when high fracture resistance is essential, potentially reducing the need for retreatments and extending the lifespan of restorations. While Microfiller and Nanofiller composites show lower fracture resistance, they may still be suitable for less demanding applications, especially where aesthetics are a priority.

In real-world clinical scenarios, while the high fracture resistance of SFRC is promising, clinicians should be cautious in directly applying these results to all cases. Individual patient factors such as bite force, oral hygiene, and specific dental anatomy should be considered. Further in vivo studies are necessary to confirm these findings, with longitudinal clinical trials providing insights into the long-term performance and durability of SFRC compared to other composite materials.

## Conclusions

The findings of the study contradict the null hypothesis and demonstrate that SFRC offers superior fracture resistance compared to nanofiller and microfiller composites. These results align with previous research indicating the effectiveness of fiber reinforcement in preventing crack propagation and providing support under high occlusal forces. While some conflicting evidence exists, highlighting the complexity of material selection, FRCs show promise as a core material for restoring endodontically treated teeth, offering improved mechanical properties that are essential for clinical success.
